# Pleiotropic effect of common *PHOX2B* variants in Hirschsprung disease and neuroblastoma

**DOI:** 10.18632/aging.101834

**Published:** 2019-02-22

**Authors:** Jinglu Zhao, Yun Zhu, Xiaoli Xie, Yuxiao Yao, Jiao Zhang, Ruizhong Zhang, Lihua Huang, Jiwen Cheng, Huimin Xia, Jing He, Yan Zhang

**Affiliations:** 1 Department of Pediatric Surgery, Guangzhou Institute of Pediatrics, Guangzhou Women and Children’s Medical Center, Guangzhou Medical University, Guangzhou 510623, Guangdong, China; 2 Department of Pediatric Surgery, The First Affiliated Hospital of Zhengzhou University, Zhengzhou 450052, Henan, China; 3 Department of Pediatric Surgery, the Second Affiliated Hospital of Xi'an Jiaotong University, Xi'an 710004, Shaanxi, China; * Equal contribution

**Keywords:** Hirschsprung’s disease, neuroblastoma, PHOX2B, pleiotropic effect

## Abstract

Hirschsprung disease (HSCR) is a heterogeneous congenital disorder that affects the enteric nervous system, while neuroblastoma is an embryonal tumor of the sympathetic nervous system. Familial cases of both HSCR and neuroblastoma appear to be functionally linked to *PHOX2B*, which plays a key role in the development of neural crest derivatives. However, the association between common *PHOX2B* variants and disease risk is contested. Additionally, large-scale examination for pleiotropy or shared genetic susceptibility in sporadic HSCR and neuroblastoma cases lacks theoretical support. Here, we report the first examination of *PHOX2B* in 1470 HSCR and 469 neuroblastoma patients with matched healthy controls. The *PHOX2B* rs28647582 polymorphism was found to be associated with HSCR (P = 2.21E-03, OR = 1.26), and each subtype of the ailment (3.22E-03 ≤ P ≤ 0.43, 1.11 ≤ OR ≤ 2.32). The association between rs28647582 and NB risk was consistent with HSCR in a recessive model, though the P value was marginal (P = 0.06). These new genetic findings indicate the potential pleiotropic effects of *PHOX2B* in both HSCR and neuroblastoma, which could guide the development of therapeutic targets for the treatment of related neurodevelopmental disorders.

## Introduction

Hirschsprung’s disease (HSCR) is a congenital disorder characterized by a partial or complete absence of ganglion cells in the nerve plexuses of the lower digestive tract. The incidence is about 1/5,000 live births in Caucasians and about 1.4/5,000 of live births in Asians, and males are 3.5-4.0 times more likely to be affected than females [1]. HSCR is divided into three subtypes – short-segment HSCR (S-HSCR), long-segment HSCR (L-HSCR) and total colonic aganglionosis (TCA) – based on the length of the intestinal segment lacking nerve cells [2,3]. As a result of extensive mutation screening in familial and sporadic patients, several genes are now known to be associated with HSCR, including *RET*, *EDNRB*, *SOX10* and *PHOX2B*, among others [4,5]. Most of those genes have been implicated in the migration, differentiation, and maturation of enteric neural crest cells, and HSCR reflects the failure of enteric neural crest-derived cells to complete their anteroposterior migration to the end of the bowel [6].

Neuroblastoma (NB) is an embryonal tumor of the sympathetic nervous system and is generally considered to originate from neural crest cells in the paravertebral sympathetic ganglia and the adrenal medulla [7]. It is the most commonly occurring extracranial cancer of early childhood, with an estimated incidence of 1/8,000-10,000 births [8]. Most NB patients are sporadic, but about 1-2% are familial cases [9]. For instance, paired mesoderm homeobox 2b gene (*PHOX2B*) and anaplastic lymphoma kinase gene (*ALK*) predict the main etiology of NB [10–12]. A subset of patients with Congenital Central Hypoventilation Syndrome (CCHS) present both HSCR and NB [12–14].

*PHOX2B* is located on chromosome 4p12 and encodes a transcription factor involved in the development of enteric neuron populations. In mice, its expression coincides with neural crest cell invasion of the foregut mesenchyme and continues throughout the process of cellular differentiation into enteric neurons [12]. In mice and zebrafish, *PHOX2B* deficiency impairs neuronal sympathetic differentiation, and results in a failure of enteric neural crest-derived cells to normally colonize the gut [15–17]. Mutant mice harboring a non-polyalanine repeat expansion mutation of *PHOX2B* exhibit the CCHS-HSCR-NB phenotype [18]. The predisposition of CCHS patients to HSCR and developmental and functional intestinal defects reflect *PHOX2B* haploinsufficiency in these patients [19].

*PHOX2B* mutations in HSCR-NB cases suggest the gene has pleiotropic effects that influence sporadic cases of both HSCR and NB [20–22]. The association between a common *PHOX2B* variant (rs28647582) and HSCR risk has been investigated in case-control studies, but the results are inconsistent [23–26]. On the other hand, *PHOX2B* mutations have been detected in a familial case of NB (mutation R100L) and in an isolated case of NB associated with HSCR (mutation R141G) [27]. Thus, much about the role of *PHOX2B* in sporadic HSCR and NB cases remains unclear. We therefore conducted a replication study of HSCR and NB cases and controls to evaluate the independent contribution of *PHOX2B* to the two ailments. Our findings offer additional insight into their etiology and provide a better understanding of the role of *PHOX2B* in sporadic HSCR and NB.

## RESULTS

### Characteristics of the study participants

Supplementary Table 1 summarizes the subclinical information for a South Chinese population that included 1470 HSCR patients and 1473 healthy controls. The age and gender distributions were similar between the cases and controls (P > 0.05). Clinical subtype manifestation and total intestine involved are listed in the table. The Supplementary Table 2 summarizes the subclinical information for a total of 469 NB patients and 998 healthy controls. Also detailed are the distribution of clinical stages and sites of tumor origin.

### *PHOX2B* and HSCR susceptibility

We initially detected a significant association between *PHOX2B* SNP rs28647582 and HSCR (P = 2.21E-03, OR = 1.26) (Table 1). To further specify the association between rs28647582 and HSCR, we classified the samples using genotypic, additive, dominant, and recessive models (7.35E-04 ≤ P ≤ 0.02; 1.21 ≤ OR ≤ 2.37) (Table 2). We observed the most significant association with disease in the recessive model (7.35E-04 ≤ P ≤ 0.02; 1.21 ≤ OR ≤ 2.37). Somewhat surprisingly, *PHOX2B* rs28647582 showed a strong association with S-HSCR, L-HSCR and TCA (3.22E-03 ≤ P ≤ 0.43; 1.11 ≤ OR ≤ 2.32), which highlights the crucial role played by *PHOX2B* rs28647582 in the disease (Table 3). By contrast, most research to date reports an impact of common variants on only one or two of the subtypes. This inconsistency may be attributable to environmental and genetic diversity among different ethnicities [4,5,28].

**Table 1:** Replication results of *PHOX2B* SNP (rs28647582) in a South Chinese population using 1470 cases and 1473 controls.

SNP	Gene	CHR	BP	A1/A2	F_A	F_U	P	OR	0.95 CI
rs28647582	*PHOX2B*	4	41747248	T/C	0.87	0.84	**2.21E-03**	1.26	(1.09~1.45)

SNP, single Nucleotide Polymorphism; CHR, chromosome; BP, base pair where the SNP is located; A1/A2, risk allele and protective allele to disease; F_A/F_U, risk allele frequency of the SNP in cases or controls; OR, odds ratio; CI, confidence interval. The P value indicates the significance based on allelic association tests. Calculation of the OR was also based on the risk allele of each SNP.

**Table 2:** Detailed replication results with different genetic models.

SNP	A1/A2	TEST	AFF	UNAFF	DF	P	OR	0.95 CI
rs28647582	*P_hwe= 0.378*							
	T/C	Genotypic	1080/346/21	1028/374/49	2	**1.13E-03**	/	/
	T/C	Additive	2506/388	2430/472	1	**2.41E-03**	1.26	(1.09~1.45)
	T/C	Dominant	1426/21	1402/49	1	**7.35E-04**	2.37	(1.42~3.98)
	T/C	Recessive	1080/367	1028/423	1	**0.02**	1.21	(1.03~1.43)

HWE, Hardy-Weinberg equilibrium; A1/A2, risk allele and protective allele to disease; AFF/UNAFF, cases and controls; DF, degree of freedom; OR, odds ratio; CI, confidence interval. The P value indicates the significance based on allelic association tests. Calculation of OR was also based on the risk allele of each SNP.

**Table 3:** The association results of *PHOX2B* gene SNP (rs28647582) to different subclinical features classified by aganglionosis length.

CHR	SNP	Length of aganglionic segment	A1/A2	F_A	F_U	P	OR
4	rs28647582	S-HSCR	T/C	0.86	0.84	**1.39E-02**	1.22(1.04~1.43)
		L-HSCR		0.85	0.84	**0.43**	1.11(0.86~1.42)
		TCA		0.92	0.84	**3.22E-03**	2.32(1.30~4.11)

SNP, single nucleotide polymorphism; CHR, chromosome; S-HSCR, short-segment HSCR; L-HSCR, long-segment HSCR; TCA, total colonic aganglionosis; A1/A2, risk allele and protective allele to disease; F_A/F_U, risk allele frequency of the SNP in cases or controls; OR, odds ratio; CI, confidence interval. The P value indicates the significance based on allelic association tests. Calculation of OR was also based on the risk allele of each SNP.

### *PHOX2B* and NB susceptibility

Genotype and allele frequencies of *PHOX2B* rs28647582 and associations with NB risk are summarized in Table 4. In both the combined and subgroup analyses, the genotype distribution of *PHOX2B* rs28647582 in the controls was consistent with Hardy-Weinberg equilibrium (P = 0.538). Although the TT and TC distributions did not significantly differ between the patients and controls, we observed a marginal difference between the CC distributions (P = 0.058). Consistent with that finding, we found a similar marginal association between the CC genotype and NB risk in the recessive model (P = 0.060). These findings suggest similar patterns of association between *PHOX2B* rs28647582 and HSCR and NB risk, suggesting a potential effect of *PHOX2B* pleiotropy in the diseases.

**Table 4:** Association of *PHOX2B* rs28647582T>C polymorphism with neuroblastoma risk.

Genotype	Cases (N=469)	Controls (N=998)	P ^a^	Crude OR (95% CI)	P	Adjusted OR (95% CI) b	P ^b^
rs28647582 (HWE=0.538)							
TT	320 (68.23)	662 (66.33)		1.00		1.00	
TC	140 (29.85)	298 (29.86)		0.97 (0.76-1.24)	0.817	0.97 (0.76-1.23)	0.794
CC	9 (1.92)	38 (3.81)		0.49 (0.23-1.03)	0.058	0.49 (0.23-1.02)	0.058
Additive			0.155	0.88 (0.72-1.08)	0.214	0.88 (0.71-1.08)	0.204
Dominant	149 (31.77)	336 (33.67)	0.471	0.92 (0.73-1.16)	0.471	0.91 (0.72-1.16)	0.454
Recessive	460 (98.08)	960 (96.19)	0.055	0.49 (0.24-1.03)	0.060	0.49 (0.24-1.03)	0.060

OR, odds ratio; CI, confidence interval; HWE, Hardy-Weinberg equilibrium.

^a^ χ^2^ test for genotype distributions between neuroblastoma patients and controls.

^b^ Adjusted for age and gender.

### Stratification analysis of *PHOX2B* rs28647582 and NB risk

To further evaluate the contributions of *PHOX2B* rs28647582 T>C polymorphisms to the risk of NB, stratification analysis based on age, gender, tumor sites of origin, and clinical stage was performed (Table 5). Although we failed to detect significant association between *PHOX2B* rs28647582 T>C polymorphism and NB risk in any of the evaluated subgroups, we observed a marginal association in patients older than 18 years of age.

**Table 5:** Stratification analysis of the association between *PHOX2B* rs28647582 T>C polymorphism and neuroblastoma risk.

Variables	Rs28647582 (cases/controls)		Crude OR	P	Adjusted OR a	P ^a^
	TT/TC	CC	(95% CI)		(95% CI)	
Age, month						
≤18	165/378	4/12	0.76 (0.24-2.40)	0.645	0.79 (0.25-2.49)	0.685
>18	295/582	5/26	0.38 (0.14-1.00)	0.050	0.38 (0.15-1.00)	0.051
Gender						
Females	192/397	4/17	0.49 (0.16-1.47)	0.200	0.49 (0.16-1.47)	0.202
Males	268/563	5/21	0.50 (0.19-1.34)	0.169	0.49 (0.18-1.33)	0.162
Sites of origin						
Adrenal gland	157/960	5/38	0.81 (0.31-2.08)	0.653	0.80 (0.31-2.06)	0.641
Retroperitoneal	135/960	3/38	0.56 (0.17-1.84)	0.341	0.56 (0.17-1.85)	0.342
Mediastinum	120/960	1/38	0.21 (0.03-1.55)	0.126	0.21 (0.03-1.52)	0.122
Others	40/960	0/38	/	/	/	/
Clinical stages						
I+II+4s	229/960	4/38	0.44 (0.16-1.25)	0.123	0.44 (0.15-1.24)	0.119
III+IV	211/960	5/38	0.60 (0.23-1.54)	0.287	0.60 (0.23-1.53)	0.283

OR, odds ratio; CI, confidence interval.

^a^Adjusted for age and gender, omitting the corresponding stratification factor.

## DISCUSSION

HSCR and NB are neurocristopathies of the sympathetic and enteric nervous systems. HSCR is an aganglionosis with a strong genetic component affecting intestinal segments of various length. A number of genes are associated with HSCR, including *PHOX2B*, haploinsufficiency of which predisposes people to defects in intestinal development and function [19,29]. Since PHOX2B was first found to be associated with HSCR in 2003, eight *PHOX2B* SNPs have been reported [24,30,31]. However, not all patient populations exhibited a significant association between *PHOX2B* and HSCR [23–26]. In the present study, we confirmed the significant association between *PHOX2B* rs28647582 and HSCR in a Southern Chinese population of 1470 cases, which is a much larger sample than in earlier studies. Moreover, our findings predict an increased risk for every subtype of HSCR.

*PHOX2B* encodes a highly conserved protein of about 314 amino acids [32]. The rs28647582 polymorphism is a variant within the intron of the gene. It is unclear whether rs28647582 directly affects *PHOX2B* expression. Our findings indicate that rs28647582 is associated with HSCR in our study population. This association may indicate a direct contribution of rs28647582 to the HSCR phenotype or that rs28647582 is a linkage disequilibrium with another susceptibility locus. Direct involvement of rs28647582 in HSCR would be through alteration of intronic sequences crucial for splicing and/or regulation of *PHOX2B* expression. In addition to SNPs, the coexistence of *RET* mutations with mutations or SNPs in *EDNRB* and *GDNF* have also been described in HSCR patients [33,34]. We therefore hypothesize that rs28647582 or other *PHOX2B* variants must occur together or act in concert with mutations in the RET and/or EDNRB signaling pathways to produce the HSCR phenotype. Remarkably, this study is the first to propose a significant link between *PHOX2B* rs28647582 and the risk of all HSCR subtypes. Relationships between HSCR subtypes and *PHOX2B* have not been reported previously, though several common genetic variants in *RET*, *EDNRB*, and *SOX10* reportedly associate with one or two of the subtypes [4,5,28]. The different HSCR subtypes reflect the severity of the intestinal neuronal precursor cell migration defect during embryonal development [35]. Our findings thus highlight the idea that dysfunction related to *PHOX2B* variants directly affect the extent of aganglionosis that arises during enteric nervous system development, ultimately leading to any of the three HSCR subtypes.

In the present study, *PHOX2B* mutations were most frequent in familial HSCR and NB cases or HSCR-NB syndrome cases [8,17]. *PHOX2B* variants had previously been identified as the major disease-causing gene in sporadic cases and in CCHS [8,11,36]. Several SNP studies examining *PHOX2B*’s association with NB in sporadic cases have been previously reported [20,37,38]; however, to our knowledge, this is the first exploration of the relationship between the *PHOX2B* 12345678 T>C polymorphism and NB risk in sporadic cases. We did not detect an association between the *PHOX2B* rs28647582 T>C polymorphism and NB risk in sporadic cases, but the trend is consistent with the findings for HSCR risk. There are several possible reasons for this negative result. First, NB mutations tend to be missense alterations in highly conserved regions or nonsense mutations that lead to a truncated protein lacking a second polyalanine motif [11,39–41]. The common genetic variants would not necessarily exert a marked influence. Second, NB is a multifactorial disease resulting from multiplicative interactions among genetic backgrounds and environmental factors. Our study lacked several valuable parameters that likely exert effects, including parental exposures, dietary intake, and living environment [42]. Third, our study includes only the rs28647582 T>C polymorphism. It is nevertheless clear that only a small fraction of SNPs influence cancer susceptibility, while most do not. Finally, there is likely some selection bias. Our study was hospital-based with subjects recruited from the provinces of Guangdong, Henan and Shaanxi. This population may not be representative of the general Chinese population.

In summary, this was the first exploration of the pleiotropic effects of common *PHOX2B* variants in HSCR and NB. Our results show that *PHOX2B* rs28647582 is independently associated with HSCR risk, but that the *PHOX2B* rs28647582 T>C polymorphism has no effect on NB risk. Nonetheless, this study has compelling biological applicability as the largest population-based study of the potential pleiotropic effects of *PHOX2B* genetic polymorphisms in HSCR and NB. Although the mechanisms remain largely undetermined, our findings provide proof-of-principle that certain genetic susceptibility loci could potentially be used to stratify individuals with respect to HSCR risk. Based on our findings, we suggest future multicenter and multiracial studies of the relation between *PHOX2B* SNPs and HSCR are warranted.

## METHODS

### Study subjects

The study included 1470 HSCR cases (age range 8.37 ± 20.50 months; 83.67% males) and 1473 controls. The patients were diagnosed with HSCR by the Pediatric Clinic of Guangzhou Women and Children’s Medical Center in Guangzhou, China between 2000 and 2015. These diagnoses were based on histologic examination of biopsy or surgical resection samples revealing the absence of enteric nerve plexuses. After dividing the cases into three subgroups based on the segment lengths exhibiting aganglionosis, there were 1033 patients with S-HSCR, 294 with L-HSCR and 82 with TCA (Supplementary Table 1). In addition, a total of 469 NB cases and 998 healthy controls were also included in this study [43]. Of those, 275 NB cases and 531 controls were from Guangzhou Women and Children’s Medical Center [44–46], 118 cases and 281 controls were from The First Affiliated Hospital of Zhengzhou University [47–49], and 76 NB cases and 186 controls were from the Second Affiliated Hospital of Xi'an Jiaotong University (Supplementary Table 2). The cases were patients diagnosed with NB, and the controls were recruited from the same hospitals. Ethical approved was obtained from the Institutional Review Board of each hospital and written informed consent was obtained from each subject.

### SNP genotyping and quality control

A common *PHOX2B* variant (rs28647582) was selected using the GTEx portal website (http://www.gtexportal.org/home/) to predict potential associations between the SNP and *PHOX2B* expression levels [50]. Past studies covering 372 cases and 511 controls have suggested that the *PHOX2B* rs28647582 genotype is not associated with HSCR [24,25,51], but we chose to re-assess *PHOX2B* rs28647582 using a much larger sample. For the 1470 HSCR cases and 1473 controls, SNPs were genotyped using a MassARRAY iPLEX Gold system (Sequenom). Hardy-Weinberg equilibrium tests were performed.

For all NB cases and controls, rs28647582 was genotyped using a TaqMan real-time PCR system on a 7900 Sequence Detection System (Applied Biosystems, Foster City, CA), as described previously [52–54]. The call rate for the SNPs was 99%, which met the pre-set criterion. For quality control, eight duplicate positive and eight negative controls without DNA were used in each 384-well plate.

### Statistical analyses

The χ^2^ test was used to evaluate differences in the frequency distributions of the demographics and genotypes between the cases and controls. A genotype test of 3 × 2 contingency tables and Cochran-Armitage trend test were used to test genotypic, additive, dominant and recessive models. These tests were carried out using PLINK software Version 1.9 [55]. SNPs were tested for associations with disease using a case comparison approach, with and without indicating the disease subtype. Odds ratios (ORs) and 95% confidence intervals (CIs) calculated using the Woolf approximation method were used to assess the correlation between the *PHOX2B* rs28647582 and HSCR and NB risk. The Hardy-Weinberg equilibrium was assessed using the goodness-of-χ^2^ test. Crude and age- and gender-adjusted ORs were assessed using the unconditional logistic regression method. SAS software (Version 9.4; SAS Institute, Cary, NC, USA) was used for these analyses. All statistical tests were two-sided. Values of P < 0.05 were considered significant.

## Supplementary Material

Supplementary Tables
