# Supplementary material for: Pleiotropic effect of common PHOX2B variants in Hirschsprung disease and neuroblastoma
Source: Aging (Albany NY). 2019 Feb 22;11(4):1252–61. doi: 10.18632/aging.101834 (PMC6402522; doi:10.18632/aging.101834)
Supplement: Supplementary Tables [file aging-11-101834-s001.pdf]

SUPPLEMENTARY TABLES

Supplementary Table 1. The subclinical information in HSCR patients collected for the subjects.

HSCR subphenotype	Cases (n=1470)		Controls (n=1473)	
	No.	%	No.	%
Subjects				
Age range（Months）	8.37±20.50		18.61±19.75	
≤2	725	49.32%	458	31.09%
>2	745	50.68%	1015	68.91%
Gender				
Females	240	16.33%	458	65.65%
Males	1230	83.67%	1015	34.35%
Clinical manifestation				
SHCSR	1033	70.27%	/	/
LHCSR	294	20.00%	/	/
TCA	82	5.58%	/	/
Total intestine	3	0.20%	/	/
Enteritis_before_operation	261	17.76%	/	/
Enteritis_after_operation	249	16.94%	/	/

Supplementary Table 2. The subclinical information in NB patients collected for the subjects.

Variables	Combined subjects					Shaanxi province				
	Cases (n=469)		Controls (n=998)		P [a]	Cases (n=76)		Controls (n=186)		P [a]
	No.	%	No.	%		No.	%	No.	%	
Age range, month	0.00-132.00		0.03-156.00		0.263	0.07-89.00		0.03-60.00		0.110
Mean ± SD	34.07±27.60		32.89±27.43			24.48±25.23		23.66±16.66		
≤18	169	36.03	390	39.08		43	56.58	85	45.70	
>18	300	63.97	608	60.92		33	43.42	101	54.30	
Gender					0.911					0.778
Female	196	41.79	414	41.48		28	36.84	72	38.71	
Male	273	58.21	584	58.52		48	63.16	114	61.29	
INSS stages										
I	133	28.42	/	/		64	84.21	/	/	
II	100	21.37	/	/		7	9.21	/	/	
III	69	14.74	/	/		1	1.32	/	/	
IV	147	31.41	/	/		4	5.26	/	/	
4s	11	2.35	/	/		/	/	/	/	
NA	8	1.71	/	/		/	/	/	/	
Sites of origin										
Adrenal gland	162	34.54	/	/		9	11.84	/	/	
Retroperitoneal region	138	29.42	/	/		51	67.11	/	/	
Mediastinum	121	25.80	/	/		12	15.79	/	/	
Other region	40	8.53	/	/		4	5.26	/	/	
NA	8	1.71	/	/		/	/	/	/	

SD, standard deviation; NA, not available.

[a] Two-sided χ^2 test for distributions between neuroblastoma cases and cancer-free controls.
